# Behind the Pain: Understanding and Treating Piriformis Syndrome

**DOI:** 10.7759/cureus.70750

**Published:** 2024-10-03

**Authors:** José F Brochado, João Pereira

**Affiliations:** 1 Physical Medicine and Rehabilitation, University Hospital Center of Algarve, Faro, Faro, PRT; 2 Famiy Medicine, Sesaram, Funchal, PRT

**Keywords:** botulinum toxin injections, conservative management, differential diagnosis, piriformis syndrome, sciatic nerve compression

## Abstract

Piriformis syndrome (PS) is a neuromuscular condition that occurs when excessive tension or anatomical variations in the piriformis muscle compress the sciatic nerve. This compression can cause pain in the buttock that radiates down the back of the affected lower limb, often resembling sciatica. This article presents a case of a 62-year-old woman with PS, characterized by left buttock pain and paresthesia along the sciatic nerve path, unresponsive to conservative treatments. Diagnosing PS is a difficult task because its symptoms often overlap with those of other conditions, such as lumbar radiculopathy and gluteal tendinopathy. Effective management requires a combination of clinical evaluation, imaging studies, and targeted treatments. Conservative management is the mainstay of treatment, with botulinum toxin injections proving effective in cases that do not respond to standard therapies. Future research should aim to refine diagnostic criteria and investigate both surgical and non-surgical treatment options.

## Introduction

Piriformis syndrome (PS) is a neuromuscular condition characterized by compression of the sciatic nerve by the piriformis muscle, which is located deep in the buttock region. The piriformis muscle, which originates from the sacrum and attaches to the greater trochanter of the femur, plays a role in external rotation and abduction of the hip. However, when this muscle becomes tight, spasms, or presents with anatomical variations, it can compress the sciatic nerve, leading to pain and neurological symptoms along the sciatic nerve pathway [1]. This compression can cause buttock pain that radiates down the posterior aspect of the ipsilateral lower limb, often mimicking the symptoms of sciatica. This similarity to sciatica, often referred to as “pseudo-sciatica,” complicates the clinical diagnosis and can lead to PS being underdiagnosed or misdiagnosed as a lumbar disc herniation or other lower back pathology [2,3].

The diagnosis of PS is primarily clinical, requiring a detailed patient history and a comprehensive physical examination. Despite this, the lack of standardized diagnostic criteria poses a significant challenge. Clinical tests such as the Freiberg test, Pace test, Beatty test, and the FAIR (flexion, adduction, internal rotation) test are used to reproduce symptoms and assess the likelihood of PS [4]. Diagnostic imaging, including magnetic resonance imaging (MRI) and electromyography (EMG), is often used to exclude other conditions, such as lumbar radiculopathy or hip joint disorders, which can present with similar symptoms [3,5]. The absence of definitive imaging findings specific to PS means that it remains a diagnosis of exclusion, often only considered after other potential causes of sciatic pain have been ruled out [6].

The treatment of PS is typically multimodal, involving conservative approaches such as physical therapy, pharmacological management, and lifestyle modifications. In cases that do not respond to these initial treatments, more invasive interventions, including image-guided corticosteroid or botulinum toxin injections, are considered [7]. Recent evidence suggests that botulinum toxin injections, which reduce muscle spasticity, may offer substantial relief in cases of refractory PS, providing a non-surgical option that can improve outcomes for patients who have not responded to conventional therapies [8].

This article presents a clinical case of PS in a 62-year-old woman and discusses various treatment options, with a particular focus on the role of botulinum toxin injections in managing refractory symptoms.

## Case presentation

A 62-year-old woman, with no significant history of lower back pain or significant trauma, presented with a 10-month history of left buttock pain. The pain was moderate (numeric pain scale = 5), exacerbated by supine and sitting positions, and relieved by standing and walking. The patient also reported paresthesia extending from the posterior aspect of the knee to the sole of the left foot, primarily occurring while seated. Previous treatment attempts, including intramuscular injections of betamethasone into the gluteus maximus and physiotherapy, had not provided significant relief.

On physical examination, the patient walked independently, showing no obvious signs of pain. Inspection of the dorso-lumbar spine revealed no notable asymmetries, and there was no pain upon percussion of the spinous processes or palpation of the paravertebral muscles. Deep palpation of the piriformis muscle elicited pain in the buttock region, while palpation of the greater trochanter did not cause pain, helping to exclude diagnoses such as trochanteric bursitis or gluteal tendinopathy.

Specific clinical tests, such as the Freiberg test (passive internal rotation of the lower limb in extension) and the FAIR test (flexion, adduction, and internal rotation), were positive, provoking pain in the buttock region. The Lasègue and Bragard tests on the left side reproduced the pain described by the patient, suggesting possible sciatic neuropathy.

**Figure 1:**
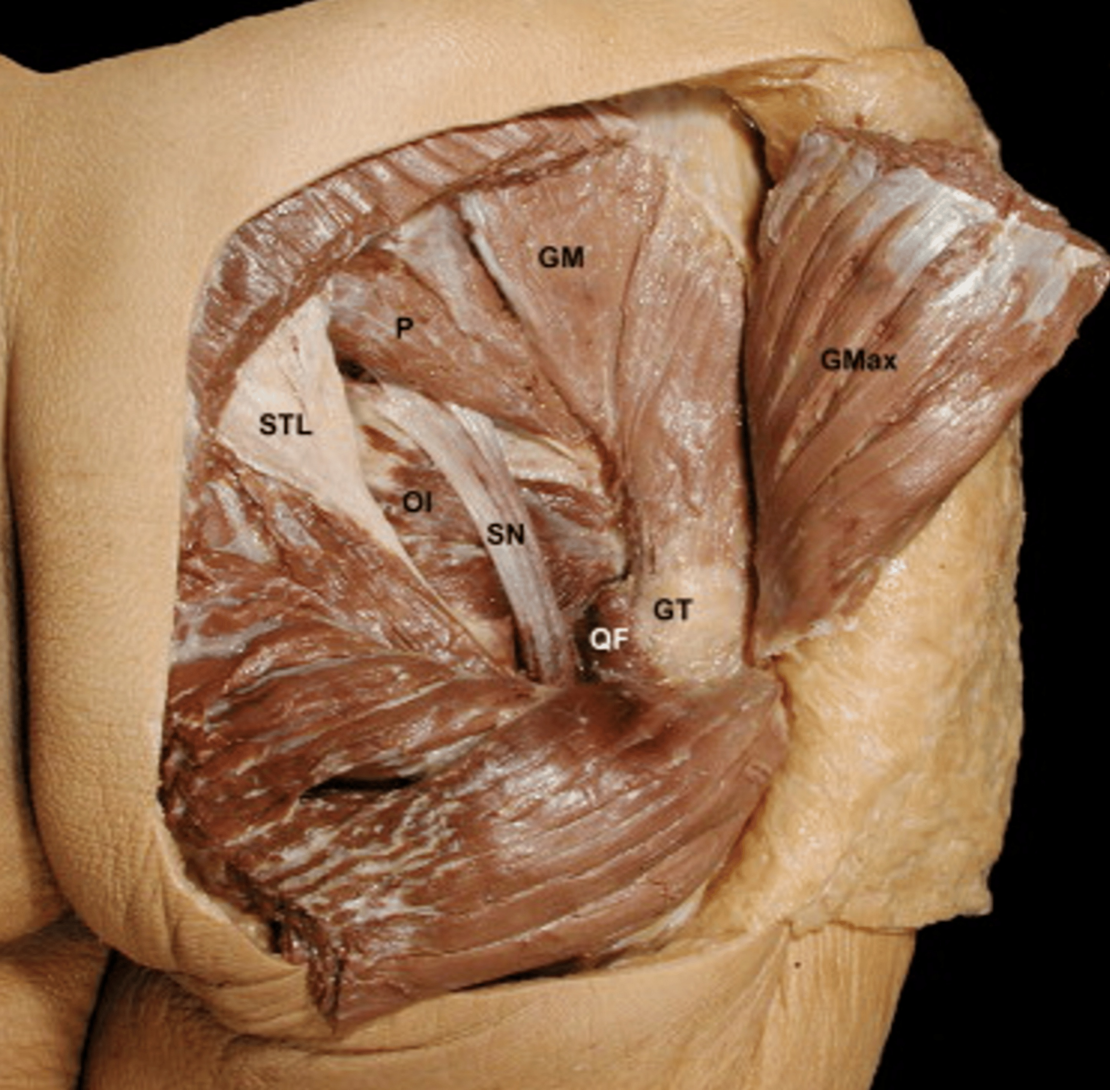
Posterior view of the right gluteal region. SN: sciatic nerve; P: piriformis muscle; GM: gluteus medius muscle; GMax: gluteus maximus muscle; OI: internal oblique muscle; QF: quadratus femoris muscle; GT: greater trochanter; STL: sacrotuberous ligament [4]

Imaging studies, including MRI and CT scans, showed an enlarged left piriformis muscle without evidence of other structural pathologies that could explain the symptoms. EMG showed signs of chronic neurogenic atrophy in the muscles associated with the left L5 myotome, suggesting irritation of the L5 nerve root. In Figure 1, we can see the close relationship between the piriformis muscle and the sciatic nerve, which could explain this pathology.

**Figure 2:**
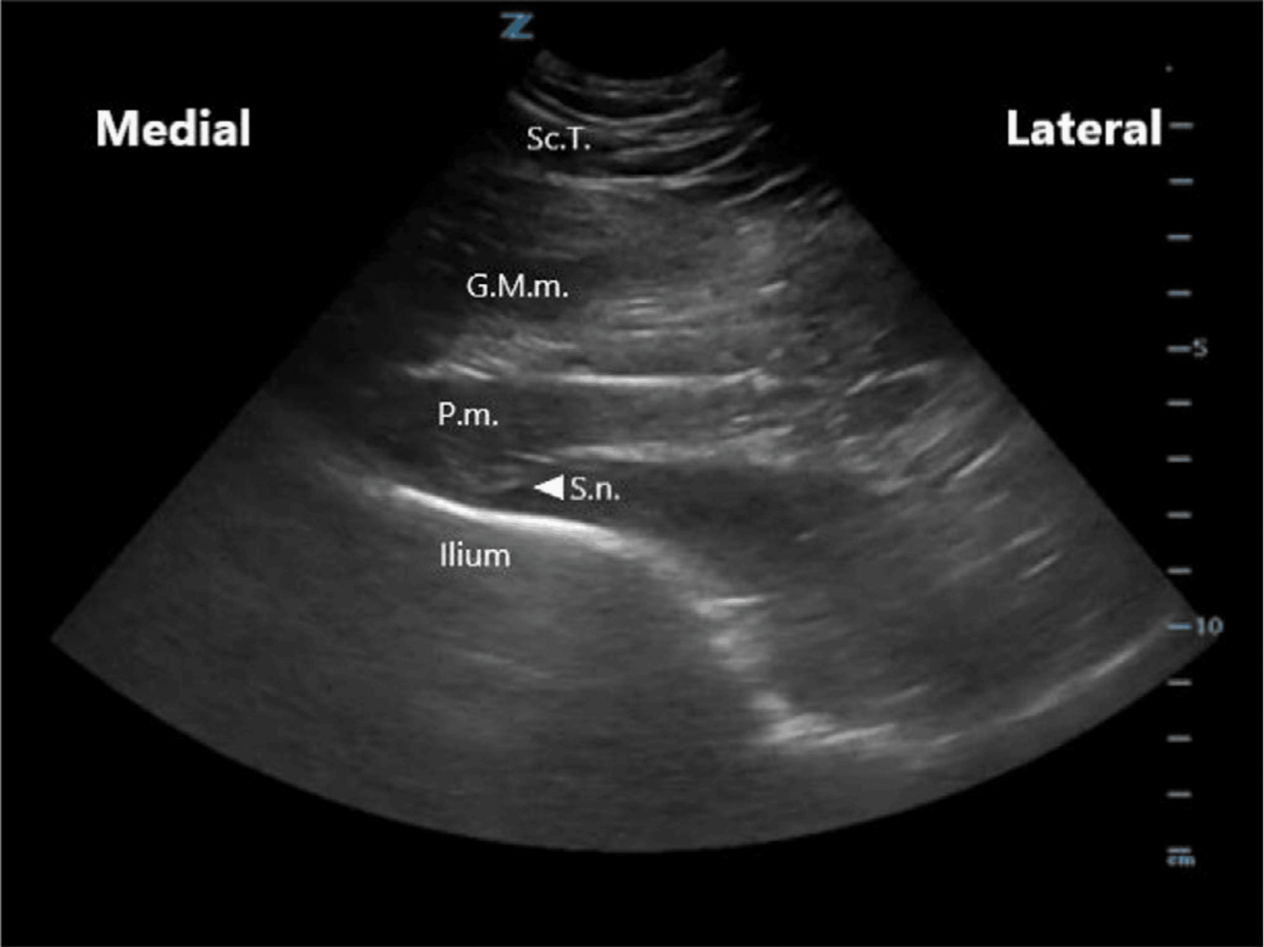
Anatomy of the piriformis muscle on ultrasound. Sc.T: subcutaneous tissue; G.M.m: gluteus maximus muscle; P.m: piriformis muscle; S.n: sciatic nerve [9].

**Figure 3:**
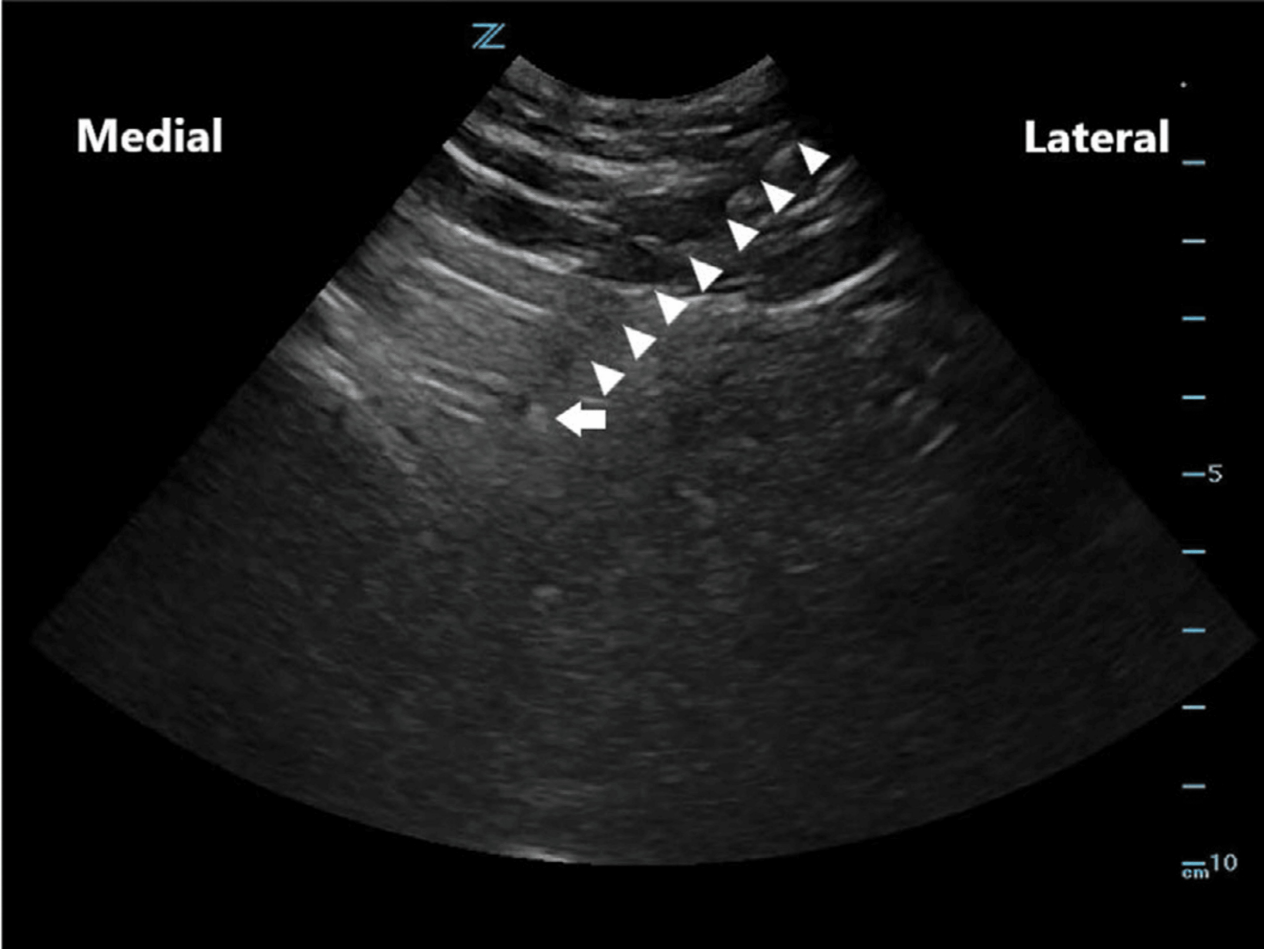
Needle visualization under ultrasound guidance. The needle is seen as it tracks through the tissues (white arrowheads) until the needle tip (white arrow) reaches the piriformis muscle [9].

Considering the clinical history, physical examination, and imaging findings, PS was the most likely diagnosis. The patient received an ultrasound-guided diagnostic injection of corticosteroid and local anesthetic into the piriformis muscle, followed by a prescribed home exercise program aimed at stretching and strengthening the piriformis muscle, improving hip flexibility, relieving pressure on the sciatic nerve, and enhancing core stability and pelvic alignment to prevent recurrence of symptoms. After four weeks, the patient reported significant improvement in symptoms, with the pain decreasing from 5 to 1 on the numeric pain scale, which supported the diagnosis of PS by confirming the piriformis muscle as the primary source of the pain. The initial injection of corticosteroid was intended as a diagnostic block, designed to temporarily relieve symptoms and verify the involvement of the piriformis muscle. Based on the positive response to this diagnostic intervention, a subsequent therapeutic injection of botulinum toxin was performed to achieve more sustained muscle relaxation, provide longer-lasting pain relief, and minimize the likelihood of symptom recurrence (Figures 2-3). The intervention was successful, and the patient remained asymptomatic during follow-up.

## Discussion

The piriformis muscle plays a crucial role in stabilizing the hip during gait and rotation of the femur, but when it becomes tense, spasmodic, or hypertrophic, it can compress the sciatic nerve, leading to a spectrum of symptoms ranging from localized buttock pain to radiating pain down the leg. This condition is particularly challenging to diagnose because its symptoms overlap significantly with other conditions, such as lumbar radiculopathy, sacroiliac joint dysfunction, and gluteal tendinopathy [1,3].

Anatomical variations in the relationship between the sciatic nerve and the piriformis muscle are common, with up to 22% of the population presenting some form of deviation from the classic anatomical description. These variations, such as a split piriformis muscle or a nerve piercing through the muscle, can predispose individuals to PS [10]. Despite these anatomical considerations, the exact pathophysiological mechanisms behind PS are still not fully understood. The muscle may compress the nerve due to hypertrophy, spasms, or changes in muscle tension, but the precise cause remains elusive in many cases [11].

The primary approach to managing PS is conservative, focusing on alleviating muscle tension and reducing nerve compression. Physical therapy, aimed at stretching and strengthening the piriformis muscle and other hip stabilizers, is a cornerstone of treatment. Non-steroidal anti-inflammatory drugs (NSAIDs) and muscle relaxants can also provide symptomatic relief [5]. In cases where conservative measures fail, image-guided injections of corticosteroids or botulinum toxin can be considered. Corticosteroid injections are often used as a diagnostic block, helping to confirm the piriformis muscle as the source of the pain by providing temporary relief. If the diagnostic injection is successful, a therapeutic injection of botulinum toxin can be administered. Botulinum toxin, in particular, reduces muscle spasticity and provides prolonged pain relief, making it an effective option for long-term management of refractory cases [7,8].

However, the variability in response to treatment and the lack of standardized diagnostic criteria highlight the need for further research. Improved imaging techniques, such as MR neurography, and a better understanding of the functional anatomy of the piriformis muscle and its relationship with the sciatic nerve, could enhance diagnostic accuracy and guide more targeted treatments [3,6].

## Conclusions

PS is a complex condition with a challenging diagnosis due to the overlap of its symptoms with other musculoskeletal conditions. The absence of standardized diagnostic criteria and specific imaging findings often leads to underdiagnosis or misdiagnosis. Conservative management remains the mainstay of treatment, focusing on physical therapy and pharmacological interventions to alleviate symptoms. In cases that do not respond to initial therapies, botulinum toxin injections have emerged as a promising non-surgical option, providing significant pain relief and improved functionality in patients with refractory PS.

Future studies should aim to refine the diagnostic criteria for PS, explore the role of advanced imaging techniques in diagnosis, and compare the long-term outcomes of surgical versus non-surgical interventions. Understanding the anatomical variations and biomechanical factors contributing to PS will be crucial in developing more effective, individualized treatment strategies.
